# Unmet Needs of Systemic Lupus Erythematosus (SLE) Patients: Insights from a Needs Assessment Study

**DOI:** 10.3390/pharmacy13050150

**Published:** 2025-10-20

**Authors:** Alyssa Ching, Annesha White

**Affiliations:** College of Pharmacy, University of North Texas Health Science Center, 3500 Camp Bowie Blvd, Fort Worth, TX 76107, USA; annesha.white@unthsc.edu

**Keywords:** needs assessment, patient education, pharmacist counseling, pharmacy education, systemic lupus erythematosus (SLE)

## Abstract

Systemic Lupus Erythematosus (SLE) is a multifaceted autoimmune disease that requires individualized care and informed patient counseling. Pharmacists are well-positioned to support SLE patients, yet gaps in knowledge and preparedness among pharmacy professionals may hinder optimal care. This study aimed to identify unmet educational and clinical needs related to SLE among pharmacy students and faculty, with the goal of informing strategies to incorporate SLE-focused education into pharmacy curricula. A systematic review of existing needs assessments was conducted, followed by a survey exploring participants’ confidence, knowledge, and perceived preparedness in managing SLE. Results revealed significant disparities in understanding and confidence, particularly among students. Faculty demonstrated moderate confidence in areas such as medication counseling and lifestyle guidance, while students reported low confidence in discussing self-care, psychosocial support, and disease management. Both groups expressed uncertainty about tools and strategies for patient education and engagement. These findings highlight educational gaps in pharmacy students’ and faculty’s preparedness to manage SLE, underscoring the need to use these insights as a foundation for curricular development. Integrating these materials into pharmacy curricula and continuing education programs can enhance pharmacists’ ability to deliver empathetic, informed, and patient-centered care. This study supports a broader effort to improve chronic disease management through interdisciplinary collaboration and educational innovation.

## 1. Introduction

Systemic Lupus Erythematosus (SLE) is a chronic autoimmune disease characterized by widespread inflammation and tissue damage affecting multiple organs, including the skin, joints, kidneys, and brain. It affects an estimated 204,000 individuals in the United States, with approximately 90% of cases occurring in women, particularly those of childbearing age [1]. SLE disproportionately impacts racial and ethnic minorities, Black, Hispanic, Asian, and American Indian/Alaska Native populations, who not only have higher prevalence rates but also tend to experience more severe disease and worse outcomes [2]. The variability in symptoms and severity among patients necessitates personalized treatment approaches to effectively manage the disease [3]. Despite advancements in medical research and treatment options, there remain significant unmet needs in the care and management of SLE patients [4]. These gaps are particularly evident in the awareness and understanding of SLE among healthcare providers, including pharmacy students and faculty. Addressing these unmet needs through comprehensive educational materials and evidence-based counseling points is crucial for improving patient outcomes and quality of life.

This study, conducted at the University of North Texas System College of Pharmacy, aimed to address these gaps by providing insights to enhance the quality of care for SLE patients through informed and empathetic pharmacy practice.

Preliminary findings from our institutional survey revealed notable disparities in confidence and preparedness between pharmacy students and faculty in managing SLE. Students reported significantly lower confidence in areas such as medication counseling, self-care guidance, and psychosocial support, with median scores ranging from 1.8 to 2.6 on a 5-point scale. Faculty demonstrated moderately higher confidence, particularly in recommending medications and lifestyle interventions, but still expressed uncertainty in patient education and engagement strategies. These gaps underscore the need for targeted curricular enhancements and serve as the foundation for this study’s focus on integrating SLE education into pharmacy training programs.

## 2. Materials and Methods

### 2.1. Study Design

To develop a tailored needs assessment for patients with SLE, a two-phase mixed-methods approach was employed. First, a targeted literature review was conducted to identify existing needs assessments and methodologies relevant to chronic disease management, with a focus on SLE. The review was conducted using PubMed and Google Scholar, with search terms including ‘systemic lupus erythematosus’, pharmacy education’, ‘patient counseling’, and ‘needs assessment’. Although the search terms were limited, they provided a foundational understanding to inform survey development. While this review informed the development of the survey instrument, it was not intended to serve as a full systematic review. We acknowledge that a comprehensive systematic review could constitute a separate manuscript. We have chosen to focus this manuscript on educational implications.

Insights from the literature review informed the development of the survey designed to capture knowledge gaps, confidence levels, and perceived preparedness among pharmacy students and faculty [5,6,7,8,9]. All pharmacy students and faculty at the University of North Texas Health Science Center were invited to complete the survey via institutional email distribution lists. No class credit, financial compensation, or other incentives were offered for participation. Participation was voluntary and anonymous.

Quantitative and qualitative data collected from the survey were analyzed to identify common themes and unmet needs. These findings were synthesized into a comprehensive SLE-specific needs assessment framework, intended to guide the development of educational interventions and pharmacist counseling strategies (Supplemental File S1).

Regarding SLE topics currently being taught in the curriculum at the University of North Texas Health Science Center College of Pharmacy, SLE is introduced within the broader context of autoimmune diseases in the Pharmacotherapy sequence, primarily during the second year. Instruction includes pathophysiology, pharmacologic treatment options, and patient counseling strategies. However, SLE-specific content is limited to a few lectures and is not reinforced through case-based learning or experiential rotations. No dedicated elective or module currently focuses exclusively on SLE or rheumatologic conditions. This limited exposure may contribute to the knowledge and confidence gaps observed in our survey.

### 2.2. Inclusion/Exclusion Criteria

Participants eligible for this study included current pharmacy students and faculty members at the University of North Texas Health Science Center who were at least 18 years of age and provided informed consent to participate. Individuals who did not meet these criteria, such as those not affiliated with the institution, under the age of 18, or who declined to participate, were excluded from the study.

### 2.3. Data Collection and Analysis

Survey data were collected using the Qualtrics online platform, which ensured secure and anonymous responses. A total of 295 individuals were invited to participate, and 10 completed the survey, yielding an overall response rate of 3.4%. Upon completion of data collection, responses were exported into Microsoft Excel for organization and preliminary analysis. Descriptive statistics were used to summarize quantitative responses, while open-ended responses were reviewed for thematic patterns. The synthesized findings were then compiled into a structured needs assessment framework to facilitate interpretation and guide the development of targeted educational materials. Table 1 summarizes survey participation and key academic characteristics of the respondents.

### 2.4. Supplementary Educational Materials

As part of this study, educational materials were developed to address the identified gaps in SLE knowledge and counseling confidence among pharmacy students and faculty. These materials include patient education handouts, pharmacist counseling checklists, and lifestyle management guides tailored to SLE. The resources were designed to be accessible, evidence-based, and culturally sensitive [10,11,12]. They are provided as Supplementary Materials and may serve as valuable tools for pharmacy educators, students, and practicing pharmacists seeking to enhance their understanding and support of SLE patients.

All educational materials provided in the Supplementary Section are original creations developed by the authors specifically for this study. These resources were informed by the survey findings and literature review and are designed to address the identified gaps in SLE-related education and counseling confidence among pharmacy students and faculty.

## 3. Results

### 3.1. Participant Demographics

**Figure 1:**
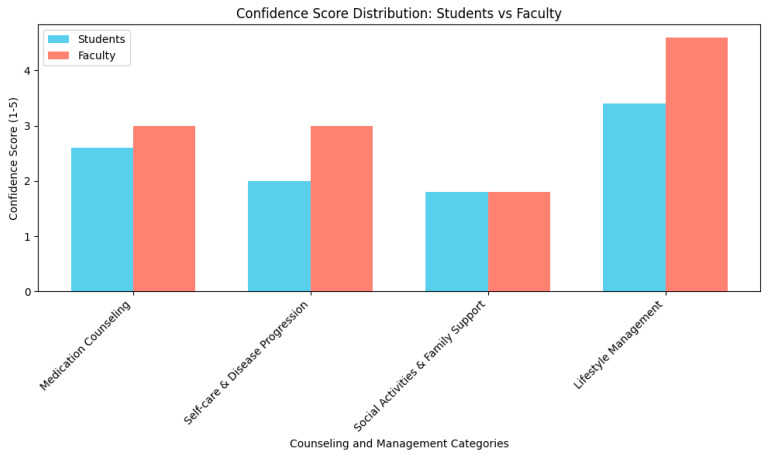
Distribution of Likert-scale responses for pharmacy students and faculty regarding confidence in SLE counseling and lifestyle management. The histogram illustrates the frequency of responses across the 5-point scale, highlighting differences in perceived preparedness between groups.

A total of 295 individuals were invited to participate in the survey, including 250 pharmacy students and 45 faculty members. Of these, 5 students and 5 faculty members completed the survey, yielding an overall response rate of 3.4%. The student respondents represented three academic years of the pharmacy program, with the majority in their second or third year. Faculty participants included individuals from diverse professional backgrounds, spanning clinical practice, administrative leadership, and pharmaceutical sciences. While demographic data such as age and gender were not collected, the sample included a range of academic levels and professional roles. This diversity in educational and occupational backgrounds provided a broad perspective on the preparedness of pharmacy professionals to support patients with SLE (Figure 1).

### 3.2. Confidence in Counseling and Management

Confidence levels varied between students and faculty. On a 5-point Likert scale, students reported low confidence in recommending medications and counseling on adverse effects, with a median score of 2.6 and an IQR of 2.24–2.97. Faculty reported moderate confidence in the same area, with a median score of 2.89 and an IQR of 2.55–3.24. Confidence in discussing self-care and disease progression was also low among students with a median score of 2.0, and only slightly higher among faculty with a median of 3.0. Both groups reported limited preparedness in educating patients on social activities and family support reflected by a shared median score of 1.8 (Table 2). Regarding perceptions of lifestyle and psychosocial support, faculty rated structured aerobic exercise as highly beneficial for SLE symptom management (median: 4.6), while students rated it as moderately beneficial (median: 3.4). Both groups agreed that SLE patients would benefit from psychosocial support for anxiety, depression, and changes in physical appearance. However, only faculty emphasized the need for support related to disease-state confusion, highlighting a broader understanding of the psychosocial complexities faced by SLE patients. Confidence in discussing self-care and disease progression was also low among students with a median score of 2.0, and only slightly higher among faculty with a median of 3.0 (Table 2). The disparities in confidence and preparedness between students and faculty, particularly in areas such as psychosocial support, point to specific curricular deficiencies. These gaps provide actionable insights for pharmacy educators seeking to enhance autoimmune disease education.

### 3.3. Awareness and Understanding of SLE

The study explored participants’ understanding of SLE symptoms, management strategies, and educational tools. While both students and faculty identified common symptoms such as rash and pain, students were less likely to mention diagnostic criteria or medication adherence. Table 3 presents categorized themes and response frequencies to clarify the distribution of knowledge and highlight areas for educational improvement.

Due to limited and uneven response distributions across survey items, statistical comparisons such as chi-square tests were attempted but were not valid for all categories. As a result, we did not include inferential statistics in this section. This limitation has been noted and considered in the interpretation of the findings.

### 3.4. Educational Gaps and Tools

Students expressed uncertainty about effective strategies for chronic pain management, tools for patient engagement, and appropriate referrals. While faculty suggested multimodal pain management (e.g., medication and cognitive behavioral therapy), students were largely unsure. Notably, none of the students identified referrals to psychological specialists, whereas faculty recommended both rheumatologists and behavioral health professionals.

Faculty identified a variety of educational tools, such as handouts, podcasts, and social media, as effective for patient education. In contrast, most students were unsure how to educate patients or what tools to use. Similarly, faculty favored educational sessions for provider support, while students lacked familiarity with such resources.

## 4. Discussion

Systemic Lupus Erythematosus (SLE) presents unique challenges in pharmacy education due to its complex symptomatology and individualized treatment needs. These findings underscore the importance of integrating SLE education into pharmacy curricula. The observed gaps in confidence and preparedness among students and faculty suggest that current training may not adequately address the complexities of autoimmune disease management. This study supports a curricular consideration toward a more case-based approach to SLE education. These gaps may serve as a foundation for curricular development, guiding the design of targeted educational interventions that address both clinical and psychosocial aspects of SLE care [13].

The low confidence reported by students in areas such as medication counseling, self-care strategies, and psychosocial support suggests a need for enhanced curricular content focused on chronic disease management. Prior studies have shown that targeted educational interventions can significantly improve pharmacy students’ clinical competence and confidence in managing complex conditions [14,15]. Faculty responses, while more confident, still reflect uncertainty in key areas, indicating that continuing professional development is equally important.

Moreover, the discrepancy between student and faculty perceptions of effective patient education tools and referral strategies underscores the importance of experiential learning. Integrating case-based learning, simulation, and patient narratives into pharmacy education may bridge these gaps and foster a more empathetic, patient-centered approach [16].

To further address these gaps, pharmacy programs can implement targeted educational interventions that go beyond traditional lecture-based instruction. Strategies such as case-based learning allow students to engage with realistic patient scenarios, fostering critical thinking and clinical decision-making. Interprofessional simulations can enhance collaboration skills and expose students to the complexities of managing chronic autoimmune conditions like SLE. Additionally, incorporating culturally sensitive educational tools, including patient narratives, multimedia resources, and inclusive counseling frameworks, can improve students’ ability to provide empathetic and personalized care.

To support these educational strategies, we developed Supplementary Materials including a pharmacist SLE education guide and a patient education handout [17,18]. These resources offer practical tools for pharmacy educators and students, such as counseling checklists, lifestyle management tips, and culturally sensitive communication strategies. These materials can help pharmacy programs to reinforce key concepts and improve student confidence in SLE care.

Given the low response rate (3.4%), the findings of this study should be interpreted as exploratory. While not generalizable, they offer preliminary insights that warrant further investigation. Future studies should include multi-institutional surveys to validate these findings and assess the broader landscape of SLE education in pharmacy programs.

We also acknowledge that SLE is likely underrepresented in most pharmacy curricula. This gap presents an opportunity for future research to explore national trends and inform curricular recommendations.

### 4.1. Proposed Pathway for SLE Education in the Pharmacy Curriculum

Based on the educational gaps identified in our study, we propose a structured pathway for integrating SLE education within the pharmacy curriculum. This pathway involves three approaches to reinforce clinical knowledge, counseling skills, and patient-centered care across all four years of training. The approach includes (1) development of an integrated patient case, (2) mapping the curriculum to SLE topics, and (3) revisiting faculty development and curriculum committee engagement to support alignment with the ACCP Pharmacotherapy Didactic Curriculum Toolkit.

According to the ACCP Pharmacotherapy Didactic Curriculum Toolkit, Systemic Lupus Erythematosus (SLE) is classified as a Tier 2 topic, indicating that foundational knowledge should be introduced during the Pharm.D. curriculum, with additional depth potentially acquired through postgraduate training or practice experience.

In recognition of this classification and the need to avoid curriculum expansion, we propose a reallocation strategy rather than adding new instructional hours. SLE content can be integrated by consolidating or replacing overlapping autoimmune disease topics (e.g., rheumatoid arthritis) within existing courses. This approach ensures that students receive targeted exposure to SLE without exceeding current curricular limits.

Regarding the integration of a case example and curricular mapping, touchpoints can be designed to fit within existing course structures, allowing for meaningful engagement with SLE concepts while maintaining curricular balance.

Specifically, the development of a longitudinal patient case example involving a young adult female newly diagnosed with SLE is included as a Supplemental File. This patient case is suggested to provide a cohesive and longitudinal learning experience that mirrors real-world patient care. For a condition like SLE, which involves fluctuating symptoms, multidisciplinary management, and psychosocial complexity, a single evolving patient case allows students to revisit and apply concepts across multiple courses and years. This approach reinforces clinical reasoning, empathy, and continuity of care. By embedding the case into various curricular touchpoints, from pathophysiology and pharmacotherapy to counseling and experiential rotations, students can gain a deeper understanding of disease progression, treatment challenges, and patient-centered strategies.

Furthermore, embedding the case into mapped curricular touchpoints, such as autoimmune pathophysiology in Year 1, pharmacologic management in Year 2, lifestyle and psychosocial support in Year 3, and experiential rotations in Year 4, students can gain a deeper understanding of disease progression, treatment challenges, and patient-centered strategies.

As an example, Year 1 integration may include the courses Introduction to Immunology and Foundations of Patient Care (2–3 h) covering the topics autoimmune pathophysiology, empathy training, and introduction to chronic illness narratives. Year 2 integration may include the Pharmacotherapy courses (4–6 h) covering the topics: SLE pharmacologic management, adverse effects, and medication adherence strategies. Year 3 integration may include the courses Patient Counseling and Public Health (3–4 h) covering the topics lifestyle management, health disparities, psychosocial support, and cultural competency. Year 4 may include the Advanced Pharmacy Practice Experiences (APPEs) integration into rheumatology, ambulatory care, or internal medicine rotations with SLE-focused patient cases.

Implementing SLE education also requires faculty development and curriculum committee engagement. Faculty can present institutional survey data to the Curriculum Committee to demonstrate the need for enhanced autoimmune disease education. Proposed changes should be aligned with ACPE standards and framed as opportunities to improve clinical competence and health equity. Pilot modules may be introduced through electives, co-curricular workshops, or interprofessional education initiatives to build support and assess feasibility.

### 4.2. Limitations

While these findings suggest actionable strategies for curricular improvement, several limitations must be considered when interpreting the results. First, the survey was conducted at a single academic institution, which may limit the generalizability of the results to other pharmacy schools or healthcare settings. The perspectives captured may not reflect the broader experiences of pharmacy students and faculty nationwide. Second, the sample size was limited, and participation was voluntary, introducing the potential for response bias. Those with a particular interest in autoimmune diseases or SLE may have been more likely to respond, potentially skewing the results. The low response rate (3.4%) is a notable limitation of this study and may affect the generalizability of the findings. The small sample size, particularly among students, means that the results may not fully represent the broader pharmacy student population. With only 10 respondents, the study lacks sufficient statistical power to draw definitive conclusions. Third, the survey relied on self-reported data, which may be subject to recall bias or social desirability bias. Participants may have over- or under-estimated knowledge and confidence levels. Finally, while the study identified key educational gaps, it did not assess the effectiveness of specific interventions. Future research should aim to increase participation to ensure more robust and representative data and include longitudinal studies to evaluate the impact of targeted educational strategies on pharmacist preparedness and patient outcomes.

In addition, we acknowledge that SLE is likely underrepresented in most pharmacy curricula, consistent with its classification as a Tier 2 topic in the ACCP Pharmacotherapy Didactic Curriculum Toolkit. This limited coverage may contribute to the gaps in confidence and preparedness observed in our survey. While our study focused on a single institution, future research could explore SLE education across multiple pharmacy programs to better understand national trends and inform broader curricular recommendations.

## 5. Conclusions

This study suggests potential opportunities to address educational and clinical gaps in the management of SLE within pharmacy education and practice. The findings reveal that both pharmacy students and faculty exhibit limited confidence and preparedness in key areas such as medication counseling, lifestyle management, and psychosocial support for SLE patients. These gaps highlight the importance of integrating evidence-based, patient-centered content into pharmacy curricula and continuing education programs. By identifying specific areas of unmet need, this study provides a foundation for developing targeted educational interventions that can enhance pharmacists’ ability to deliver comprehensive and empathetic care.

Future efforts should prioritize the integration of SLE education into pharmacy curricula through innovative teaching strategies such as case-based learning. These approaches can strengthen pharmacist competencies in SLE care and ensure that pharmacy graduates are equipped to meet the needs of this patient population. To support this goal, we have included example educational materials as Supplementary Content, which may serve as valuable resources for pharmacy students, faculty, and patients alike. These materials are intended to facilitate learning, improve communication, and promote more effective patient engagement.

As a result of integrating case-based learning and culturally responsive materials into courses, pharmacy programs can better prepare students to manage SLE and other complex chronic conditions. These approaches not only enhance clinical competence but also promote patient-centered care and health equity.

## Figures and Tables

**Table 1:** Survey Summary Table.

Participant Group	Number Invited	Number Responded	Response Rate (%)	Key Demographic Notes
Students	250	5	2.0	Three academic years represented
Faculty	45	5	11.1	Clinical and academic specialties

**Table 2:** Confidence levels of pharmacy students and faculty in SLE counseling and lifestyle management (Median and IQR).

Survey Item	Group	Median Score	IQR	Key Takeaways
Confidence in recommending medications and counseling	Students	2.6	2.2–3.0	Faculty feel somewhat confident in recommending appropriate medications and counseling, while students feel less confident with recommending medication and counseling SLE patients.
Confidence in recommending medications and counseling	Faculty	3.0	2.7–3.3	
Confidence in discussing self-care and disease progression	Students	2.0	1.8–2.3	60% of students are not confident in discussing self-care and SLE management.
Confidence in discussing self-care and disease progression	Faculty	3.0	2.6–3.4	
Preparedness in educating on social activities and family support	Students	1.8	1.5–2.1	Students and Faculty feel slightly prepared to educate SLE patients on social activities and educating their family and friends with helping SLE patients cope.
Preparedness in educating on social activities and family support	Faculty	1.8	1.6–2.0	
Perceived benefit of structured aerobic exercise for SLE symptom management	Students	3.4	3.0–3.8	Faculty find aerobic exercise extremely beneficial to SLE patients. In comparison, students found aerobic exercise somewhat beneficial.
Perceived benefit of structured aerobic exercise for SLE symptom management	Faculty	4.6	4.3–4.9	

Note: Median and IQR values are calculated using numerical representations of Likert scale responses (1–5). Decimal values may appear due to small sample sizes and the nature of median/IQR calculations, which can result in midpoints between ordinal categories.

**Table 3:** Categorized Themes and Response Frequencies.

Theme	Keywords	Response Frequency Students	Response Frequency Faculty
Symptoms	rash, pain, inflammation, fatigue	5	5
Management	diagnostic criteria, medication safety, adherence	2	2
Education Tools	handouts, podcasts, social media, sessions	1	4

## Data Availability

The original contributions presented in this study are included in the article/Supplementary Material. Further inquiries can be directed to the corresponding author.

## Supplementary Materials

The following supporting information can be downloaded at: https://www.mdpi.com/article/10.3390/pharmacy13050150/s1, File S1: Pharmacist SLE Education and Awareness—A resource for pharmacy educators and students to support curriculum development and counseling training; File S2: Patient SLE Education Handout—A culturally sensitive tool for use in patient education and experiential learning settings. File S3: Integrated Case Example for SLE Education in Pharmacy Curriculum. These materials are intended to support pharmacy education and enhance patient engagement in SLE management.
