# Supplementary material for: Unmet Needs of Systemic Lupus Erythematosus (SLE) Patients: Insights from a Needs Assessment Study
Source: Pharmacy (Basel). 2025 Oct 20;13(5):150. doi: 10.3390/pharmacy13050150 (PMC12567102; doi:10.3390/pharmacy13050150)
Supplement: Supplementary file 1 [file pharmacy-13-00150-s001.zip › S2 Patient SLE Education Handout.pdf]

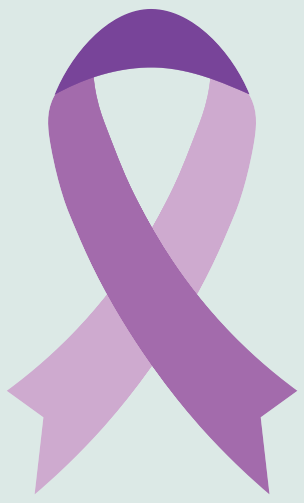

SYSTEMIC LUPUS ERYTHEMATOSUS (SLE)

What is Lupus?

Lupus, also known as Systemic Lupus Erythematosus (SLE), is a chronic autoimmune disease where the immune system attacks healthy tissues, causing inflammation and damage to various parts of the body, including the skin, joints, kidneys, heart, lungs, and brain.

Symptoms of Lupus

- Fatigue
- Joint pain and swelling
- Skin rashes, including a butterfly-shaped rash across the cheeks and nose
- Fever
- Hair loss
- Sensitivity to sunlight
- Chest pain when taking deep breaths
- Mouth sores
- Swollen lymph nodes

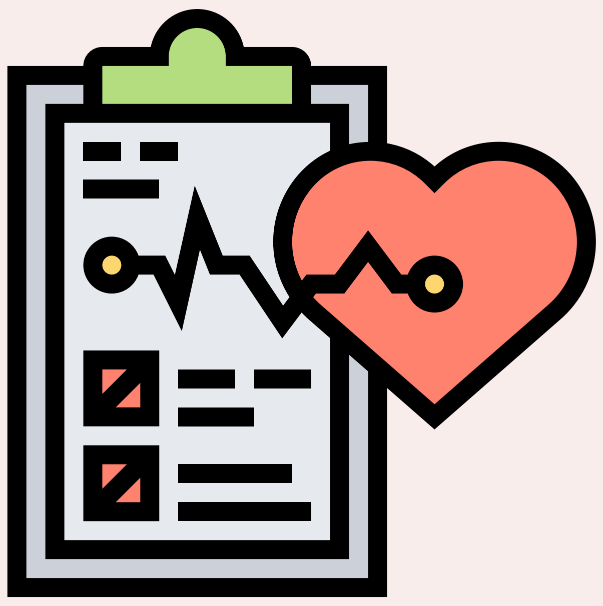

Diagnosis

Diagnosing lupus can be challenging as its symptoms often mimic those of other diseases. A combination of blood tests, urine tests, and clinical evaluation by a healthcare provider is typically used to diagnose lupus.

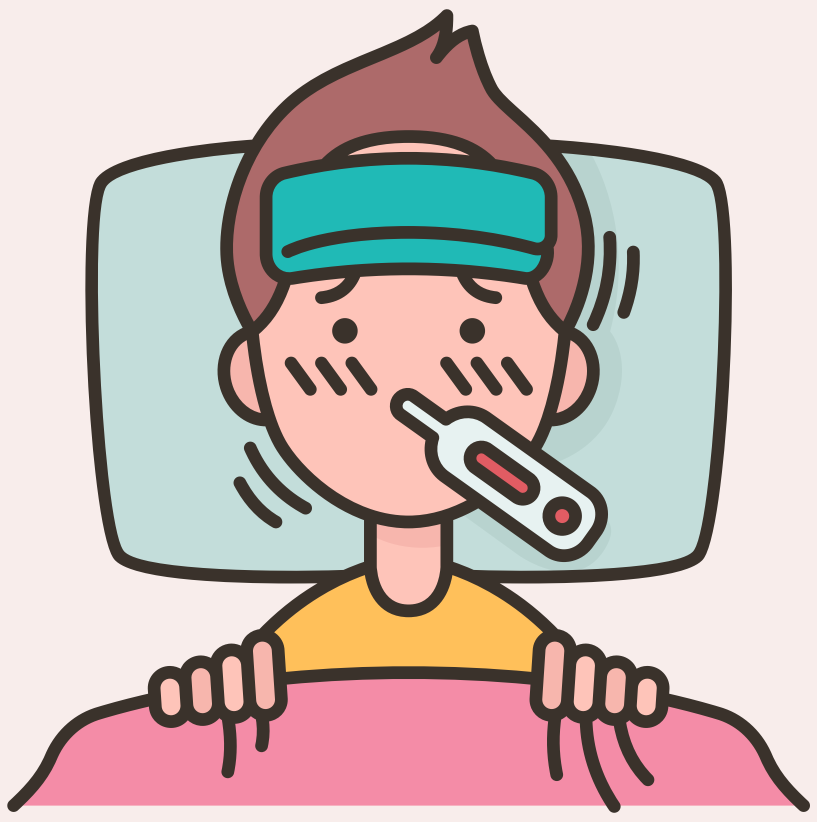

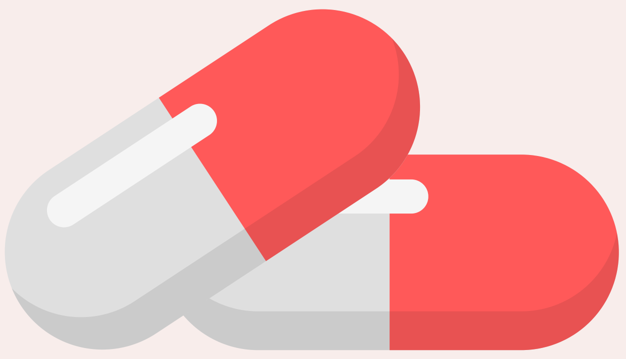

Medications

- Nonsteroidal anti-inflammatory drugs (NSAIDs) for pain and inflammation
- Antimalarial drugs to reduce flare-ups
- Corticosteroids to control inflammation
- Immunosuppressive drugs to reduce immune system activity
- Biologics to target specific parts of the immune system

Treatment

While there is no cure for lupus, treatments can help manage symptoms and prevent flare-ups.

For more information or support, please contact your healthcare provider or the Lupus Foundation of America at 1-800-558-0121.

Living with Lupus

Living with lupus can be challenging, but with proper management and support, many people with lupus lead fulfilling lives. It's important to work closely with your healthcare team, stay informed about your condition, and reach out to support groups for emotional and practical support.

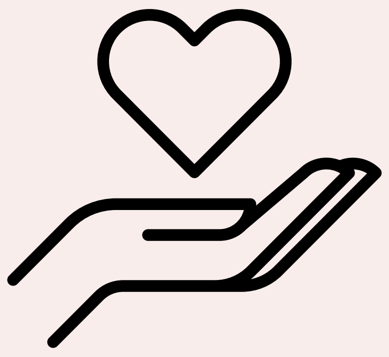

Self-Care Tips

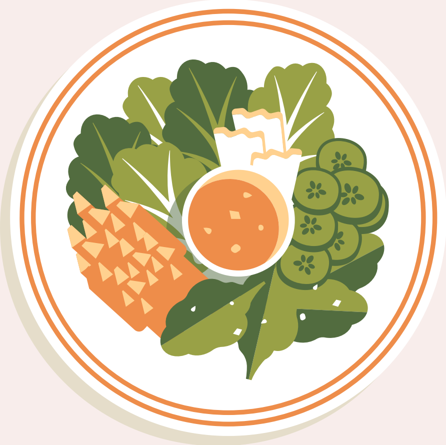

1. Healthy Diet

Eat a balanced diet rich in fruits, vegetables, whole grains, and lean proteins. Some people with lupus find that avoiding alfalfa sprouts and highly processed foods helps them manage their symptoms.

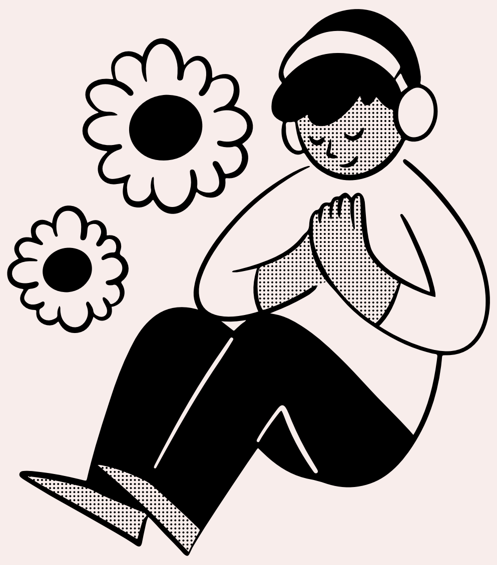

2. Rest and Pace Yourself

Fatigue is a common symptom of lupus. Ensure you get enough rest and avoid overexertion.

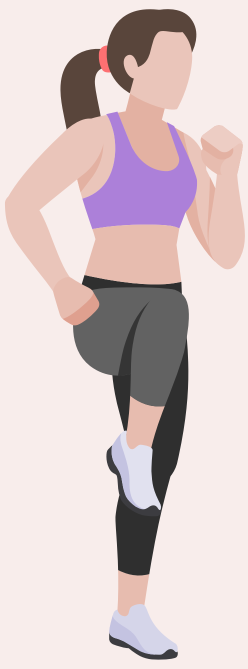

3. Regular Exercise

Engage in low-impact exercises like walking, swimming, or yoga to maintain joint flexibility and overall health.

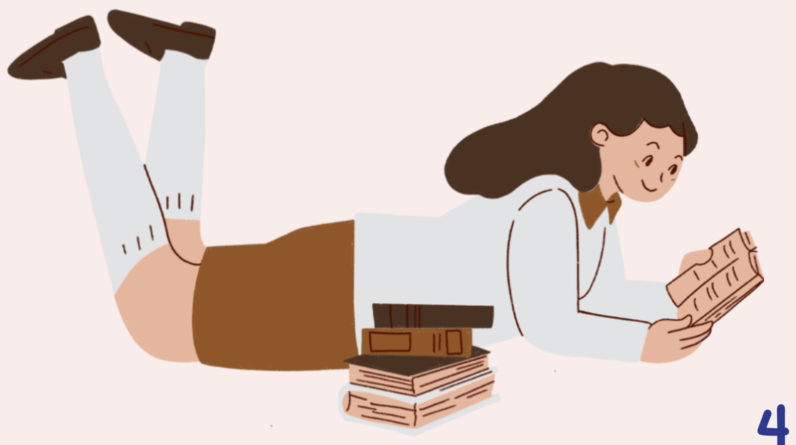

4. Stress Management

Practice stress-reducing techniques such as meditation, deep breathing exercises, and hobbies you enjoy.

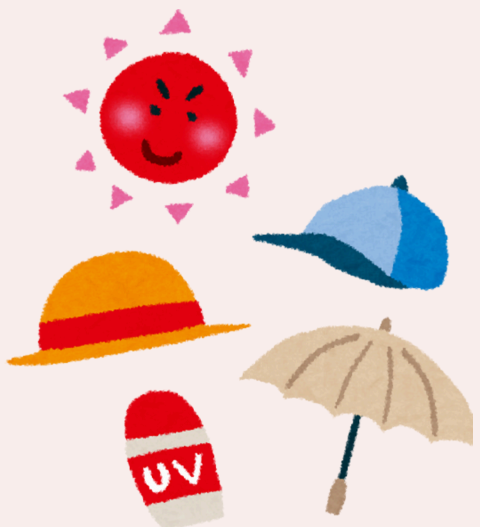

5. Sun Protection

Wear sunscreen and protective clothing to prevent skin rashes and flare-ups caused by sun exposure.

Resources

- Lupus Foundation of America: www.lupus.org
- National Institute of Arthritis and Musculoskeletal and Skin Diseases: www.niams.nih.gov
- American College of Rheumatology: www.rheumatology.org

For more information or support, please contact your healthcare provider or the Lupus Foundation of America at 1-800-558-0121.
