# Supplementary material for: Unmet Needs of Systemic Lupus Erythematosus (SLE) Patients: Insights from a Needs Assessment Study
Source: Pharmacy (Basel). 2025 Oct 20;13(5):150. doi: 10.3390/pharmacy13050150 (PMC12567102; doi:10.3390/pharmacy13050150)
Supplement: Supplementary file 1 [file pharmacy-13-00150-s001.zip › S1 Pharmacist SLE Education and Awareness.pdf]

SYSTEMIC LUPUS ERYTHEMATOSUS (SLE)

Pharmacists can play a significant role in managing the psychological impacts of lupus by providing support, education, and resources to patients.

Comprehensive Patient Education

- Provide detailed information about lupus, its symptoms, and treatment options. Use pamphlets, brochures, and other educational materials to help patients understand their condition.
- Educate patients on the importance of medication adherence and the potential side effects of their treatments.

Multidisciplinary Approach

- Collaborate with a team of healthcare professionals, including rheumatologists, dermatologists, nephrologists, and mental health specialists, to provide comprehensive care for lupus patients.
- Encourage patients to seek support from dietitians, physical therapists, and social workers as needed.

Regular Monitoring and Follow-Up

- Schedule regular follow-up appointments to monitor the patient's condition and adjust treatment plans as necessary.
- Conduct routine blood tests and other diagnostic tests to track disease activity and organ function.

Lifestyle and Self-Care

- Advise patients on lifestyle changes that can help manage their symptoms, such as maintaining a healthy diet, engaging in regular exercise, and avoiding triggers like sunlight.
- Encourage patients to practice stress management techniques, such as meditation, yoga, and deep breathing exercises.

Patient-Centered Communication

- Foster open and empathetic communication with patients. Listen to their concerns and involve them in decision-making about their treatment plans.
- Provide verbal counseling tips to guide conversations with patients, covering key points like medication adherence, lifestyle changes, and coping strategies.

Addressing Mental Health

- Recognize the psychological impact of lupus on patients and provide resources for mental health support.
- Refer patients to mental health professionals for counseling and therapy if needed.

For more information, visit the Lupus Foundation of America at www.Lupus.org or call 1-800-558-0121.

Pharmacist Responsibilities

Pharmacists can play a crucial role in improving the care and quality of life for patients with Lupus. It is important for pharmacists to stay up-to-date with guidelines and new medications.

Medication Management

- Review Medication Regimens: Regularly review patients' medication regimens to ensure they are receiving the most effective and appropriate treatments. Pay attention to potential drug interactions and adverse effects.
- Monitor Adherence: Encourage patients to adhere to their prescribed medication regimens. Use tools like pill organizers, reminder apps, and follow-up calls to support adherence.

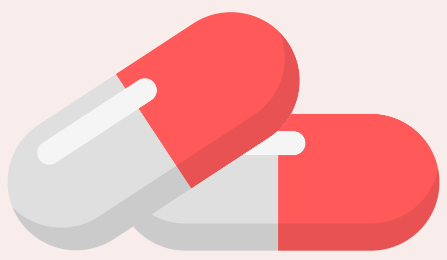

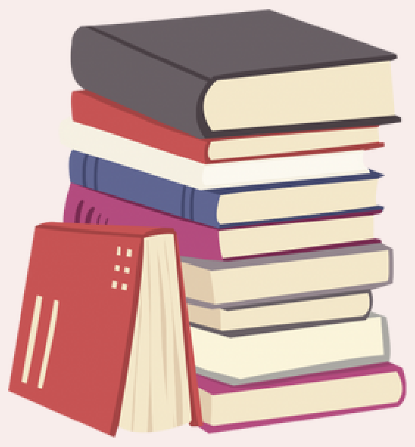

Continuing Education

- Stay Informed: Keep up-to-date with the latest research and guidelines on lupus management. Participate in continuing education programs and workshops to enhance your knowledge and skills.
- Educate Peers: Share your knowledge with other pharmacists and healthcare providers. Provide training and resources to help them better understand and manage lupus.

Disease Management

- Symptom Monitoring: Encourage patients to keep track of their symptoms and any changes in their condition. This can help in identifying flare-ups early and adjusting treatment plans accordingly.
- Lifestyle Recommendations: Advise patients on lifestyle changes that can help manage their symptoms, such as maintaining a healthy diet, engaging in regular exercise, and avoiding triggers like sunlight.

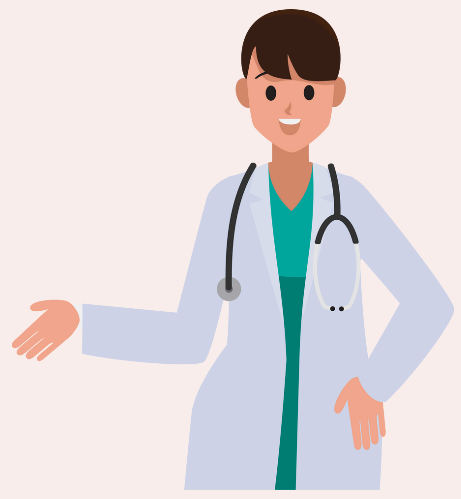

Patient Education

- Provide Clear Instructions: Ensure patients understand how to take their medications correctly, including dosage, timing, and any special instructions (e.g., taking with food).
- Educate on Side Effects: Inform patients about potential side effects of their medications and what to do if they experience them. Provide guidance on managing minor side effects and when to seek medical attention.

Collaboration with Healthcare team

- Communicate with Providers: Maintain open communication with other healthcare providers involved in the patient's care. Share relevant information about the patient's medication regimen and any concerns or observations.
- Participate in Multidisciplinary Teams: Work as part of a multidisciplinary team to provide comprehensive care for lupus patients. Collaborate with rheumatologists, dermatologists, nephrologists, and mental health specialists.

Support and Resources

- Provide Support: Offer emotional support to patients and their families. Listen to their concerns and provide reassurance and encouragement.
- Connect to Resources: Help patients access additional resources, such as support groups, educational materials, and financial assistance programs.

For more information, visit the Lupus Foundation of America at www.Lupus.org or call 1-800-558-0121.

Managing Lupus Flare-ups

Pharmacists can play a significant role in managing the physical and psychological impacts of Lupus by providing medication, support, education, and resources to patients.

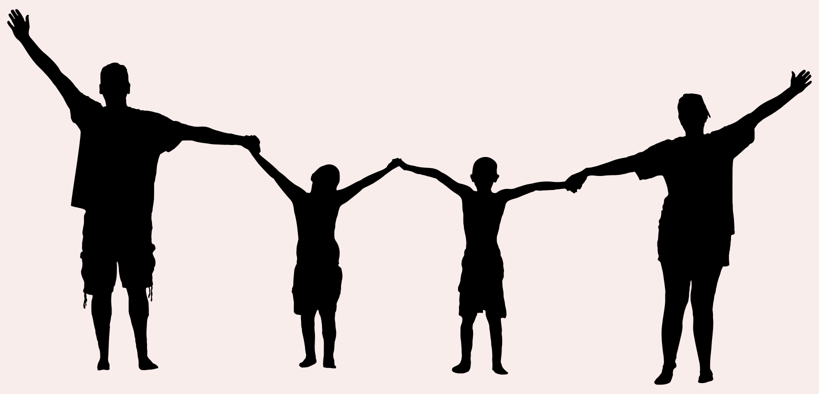

Support and Monitoring

- Regular Check-Ins: Pharmacists can schedule regular check-ins with patients to monitor their condition and provide ongoing support. These check-ins can help identify early signs of flare-ups and allow for timely intervention.
- Collaborate with Healthcare Providers: Pharmacists can work closely with other healthcare providers, such as rheumatologists and primary care physicians, to ensure a coordinated approach to managing lupus. They can share relevant information about the patient's medication regimen and any concerns or observations.

Medication Management

- Review and Adjust Medications: Pharmacists can review patients' medication regimens to ensure they are taking the correct medications and dosages. They can also identify potential drug interactions and side effects that may contribute to flare-ups.
- Monitor Adherence: Ensuring that patients adhere to their prescribed medication regimens is essential. Pharmacists can provide tools like pill organizers and reminder apps to help patients stay on track.
- Provide Medication Counseling: Pharmacists can educate patients on how to take their medications correctly, including the importance of taking them consistently and as prescribed.

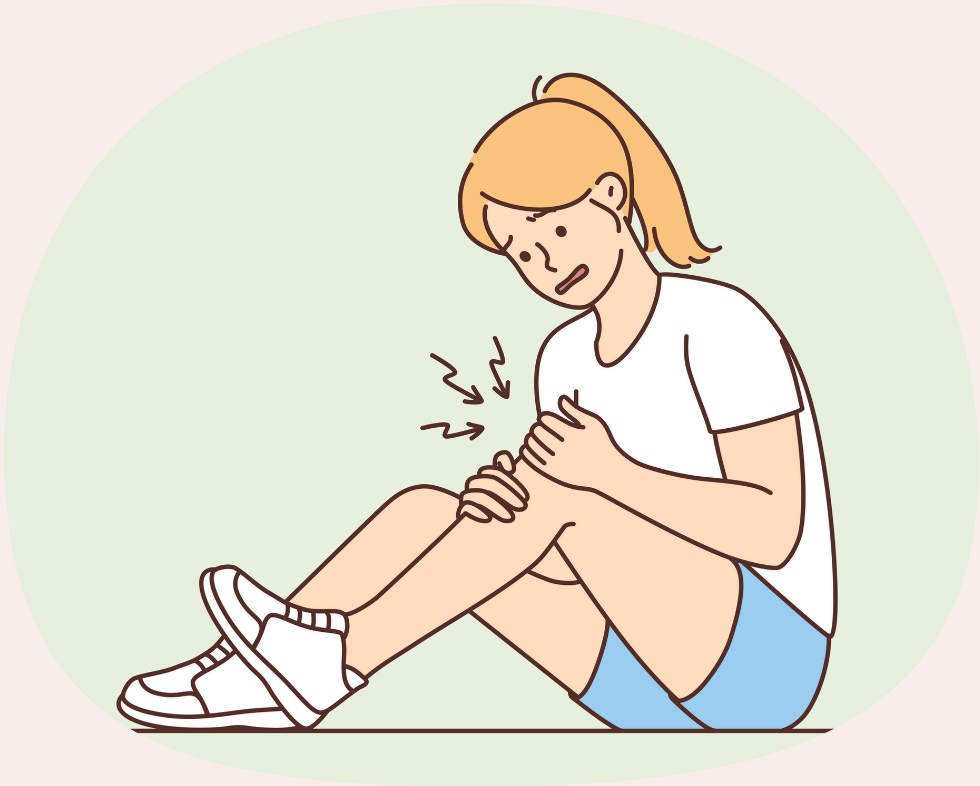

Patient Education

- Educate on Flare-Up Triggers: Pharmacists can inform patients about common triggers for lupus flare-ups, such as stress, infections, and sun exposure. They can provide tips on how to avoid these triggers and manage their symptoms.
- Offer Self-Care Tips: Pharmacists can advise patients on lifestyle changes that can help manage their symptoms, such as maintaining a healthy diet, engaging in regular exercise, and practicing stress management techniques.
- Provide Resources: Pharmacists can offer educational materials, such as pamphlets and brochures, that explain lupus and its management. They can also direct patients to support groups and online resources for additional information and support.

Emergency Preparedness

- Prepare for Flare-Ups: Pharmacists can help patients develop a plan for managing flare-ups, including having an emergency supply of medications and knowing when to seek medical attention.
- Educate on Emergency Medications: Pharmacists can educate patients on the use of emergency medications, such as corticosteroids, that may be prescribed for severe flare-ups. They can explain how to use these medications correctly and what to expect.

For more information, visit the Lupus Foundation of America at www.Lupus.org or call 1-800-558-0121.

Medication Management

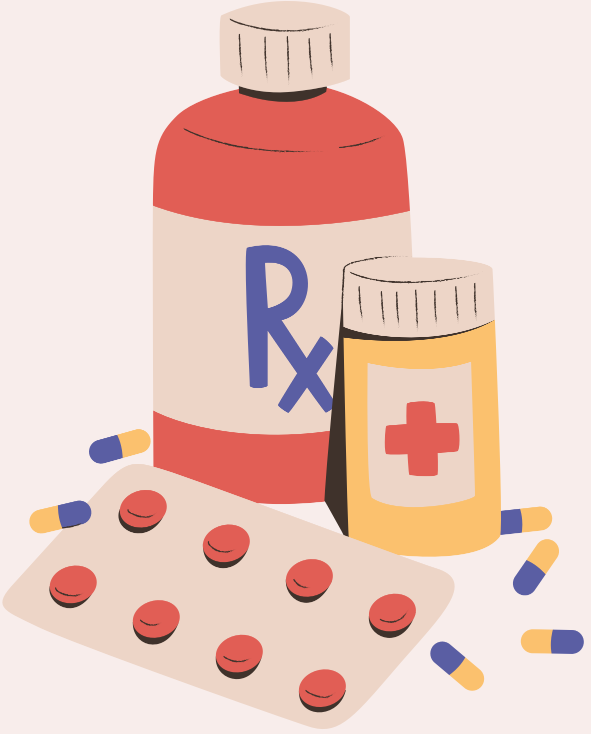

Pharmacists should be vigilant about several common drug interactions in patients with Lupus.

Nonsteroidal Anti-Inflammatory Drugs (NSAIDs) and Anticoagulants

NSAIDs, commonly used to manage pain and inflammation in lupus patients, can increase the risk of bleeding when taken with anticoagulants like warfarin. This interaction can lead to serious bleeding complications.

Antimalarial Drugs and Cardiac Medications

Hydroxychloroquine, an antimalarial drug used to manage lupus symptoms, can interact with cardiac medications like digoxin. This interaction can increase digoxin levels, leading to toxicity.

Proton Pump Inhibitors (PPIs) and Methotrexate

PPIs, used to manage gastrointestinal symptoms, can interact with methotrexate, increasing its levels and the risk of toxicity.

Antibiotics and Methotrexate

Certain antibiotics, such as trimethoprim-sulfamethoxazole, can interact with methotrexate, increasing the risk of methotrexate toxicity. This can lead to bone marrow suppression and other serious side effects.

Corticosteroids and Immunosuppressants

Corticosteroids, such as prednisone, are often prescribed to reduce inflammation. When combined with immunosuppressants like methotrexate or azathioprine, there is an increased risk of immunosuppression, making patients more susceptible to infections.

Biologics and Live Vaccines

Biologic agents, such as rituximab or belimumab, used in lupus treatment can interact with live vaccines. Patients on biologics should avoid live vaccines due to the risk of infection.

Calcium Channel Blockers and Immunosuppressants:

Calcium channel blockers, used to manage hypertension, can interact with immunosuppressants like cyclosporine. This interaction can increase cyclosporine levels, leading to nephrotoxicity.

For more information, visit the Lupus Foundation of America at www.Lupus.org or call 1-800-558-0121.

Pharmacist Role and Mental Health

Pharmacists can play a significant role in managing the psychological impacts of lupus by providing support, education, and resources to patients. Here are some ways pharmacists can help:

Providing Emotional Support

Pharmacists can offer a listening ear and provide emotional support to lupus patients. By showing empathy and understanding, pharmacists can help patients feel heard and validated, which can alleviate some of the psychological burden associated with the disease.

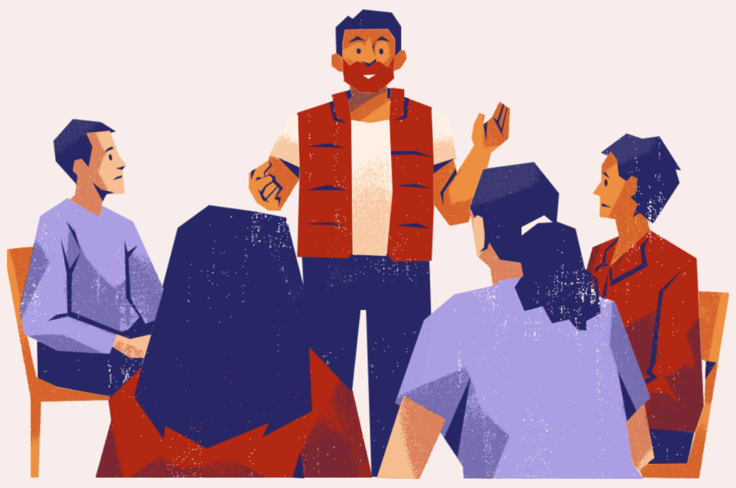

Encouraging Self-Care and Stress Management

Pharmacists can advise patients on self-care practices and stress management techniques that can help improve their mental health. This includes recommending activities such as exercise, meditation, and hobbies that can reduce stress and promote well-being.

Educating Patients

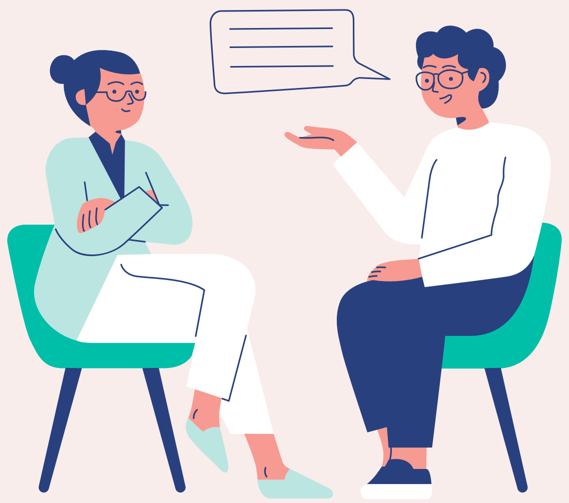

Pharmacists can educate patients about lupus and its psychological impacts. This includes explaining how the disease can affect mental health and providing information on coping strategies. Pharmacists can also educate patients on the importance of adhering to their treatment plans to manage both physical and psychological symptoms.

Identifying and Addressing Medication Side Effects

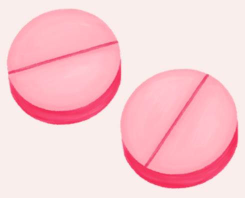

Some medications used to treat lupus can have psychological side effects, such as mood swings or depression. Pharmacists can monitor patients for these side effects and work with healthcare providers to adjust treatment plans if necessary. They can also provide information on managing these side effects and refer patients to mental health professionals if needed.

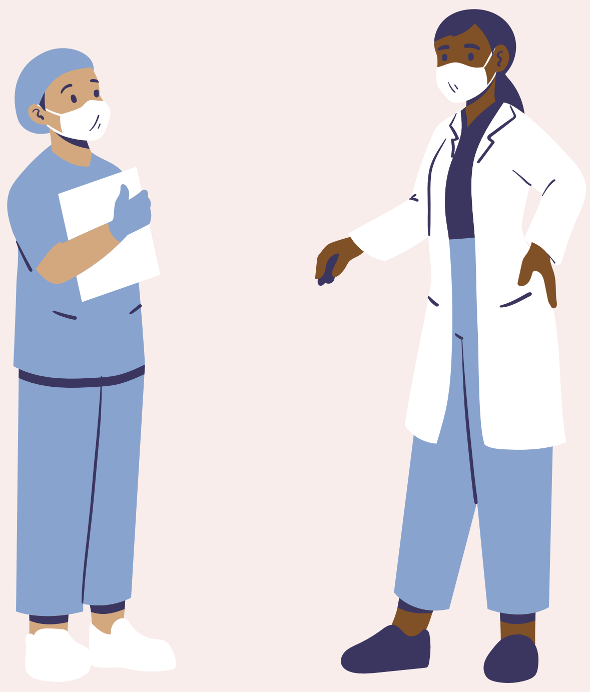

Collaborating with Healthcare Providers

Pharmacists can work closely with other healthcare providers, such as rheumatologists and mental health professionals, to ensure a comprehensive approach to managing lupus. By sharing information and collaborating on treatment plans, pharmacists can help address both the physical and psychological aspects of the disease.

By providing these services, pharmacists can help lupus patients manage the psychological impacts of the disease and improve their overall quality of life.

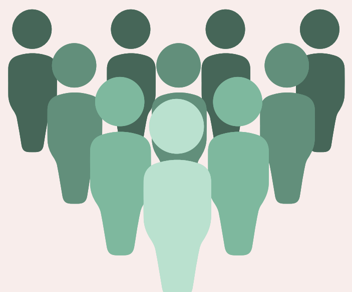

Providing Resources and Referrals

Pharmacists can connect patients with resources and support groups that can help them cope with the psychological impacts of lupus. This includes referring patients to mental health professionals, such as psychologists or counselors, and providing information on local or online support groups.

For more information, visit the Lupus Foundation of America at www.Lupus.org or call 1-800-558-0121.

Resources

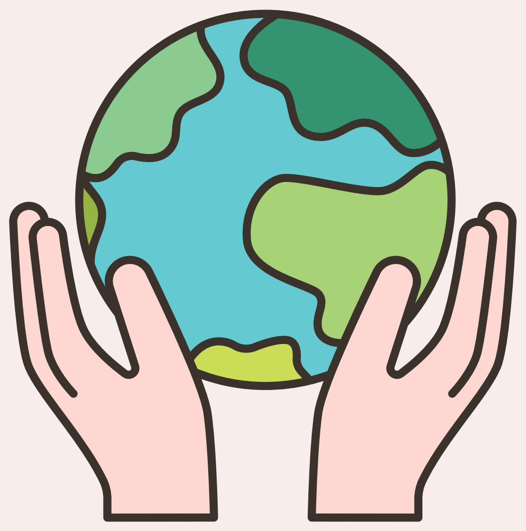

Support groups and resources for lupus patients can provide valuable support and information. Patients can learn more about their medical condition or can connect with others who understand their experiences and challenges.

Lupus Foundation of America:

This organization offers a variety of support groups, both in-person and online, for lupus patients and their families. They provide resources, educational materials, and opportunities to connect with others who are living with lupus. You can find more information on their website: www.lupus.org.

MyLupusTeam

This is a social network specifically for people living with lupus. It allows members to connect, share experiences, and gain practical advice on managing lupus. You can join the community and participate in discussions, ask questions, and find emotional support from others who understand what you're going through.

National Institute of Arthritis and Musculoskeletal and Skin Diseases (NIAMS):

NIAMS provides information and resources for lupus patients, including support groups and educational materials. Their website offers a wealth of information on managing lupus and finding support: www.niams.nih.gov.

American College of Rheumatology (ACR)

The ACR offers resources and support for lupus patients, including information on finding a rheumatologist and connecting with support groups. Their website is a valuable resource for patients and healthcare providers: www.rheumatology.org.

LupusConnect

Hosted by the Lupus Foundation of America, LupusConnect is an online community where individuals with lupus and their loved ones can engage with others to share experiences, find emotional support, and discuss practical insights for coping with the daily challenges of the disease.

For more information, visit the Lupus Foundation of America at www.Lupus.org or call 1-800-558-0121.
