# Supplementary material for: Unmet Needs of Systemic Lupus Erythematosus (SLE) Patients: Insights from a Needs Assessment Study
Source: Pharmacy (Basel). 2025 Oct 20;13(5):150. doi: 10.3390/pharmacy13050150 (PMC12567102; doi:10.3390/pharmacy13050150)
Supplement: Supplementary file 1 [file pharmacy-13-00150-s001.zip › S3 Integrated Case Example for SLE Education in Pharmacy Curriculum.pdf]

S3 Supplemental File: Integrated Case Example for SLE Education in Pharmacy Curriculum

This supplemental file presents a longitudinal case example designed to support the integration of Systemic Lupus Erythematosus (SLE) education across the pharmacy curriculum. The case follows a young adult female patient newly diagnosed with SLE and is revisited throughout the four years of pharmacy training to reinforce clinical decision-making, counseling, and psychosocial support. The patient described in this file is entirely fictional. Any similarity to actual persons, living or dead, is purely coincidental. No real patient data was used.

Patient Case Overview
Patient: Jane Doe, 24-year-old Hispanic female
Diagnosis: Newly diagnosed with Systemic Lupus Erythematosus (SLE)
Presenting Symptoms: Fatigue, joint pain, facial rash, photosensitivity
Medical History: Mild hypertension, family history of autoimmune disease
Social History: Lives alone, works full-time, limited access to healthcare resources

Curricular Mapping and Integration
Year 1: Introduction to Immunology and Foundations of Patient Care (2–3 hours)
- Topics: Autoimmune pathophysiology, chronic illness narratives, empathy training
- Case Use: Introduction to Jane's diagnosis and psychosocial context

Year 2: Pharmacotherapy (4–6 hours)
- Topics: SLE pharmacologic management, adverse effects, medication adherence
- Case Use: Jane begins treatment with hydroxychloroquine and corticosteroids; students evaluate therapy options and side effect profiles

Year 3: Patient Counseling and Public Health (3–4 hours)
- Topics: Lifestyle management, health disparities, psychosocial support, cultural competency

- Case Use: Jane experiences medication fatigue and emotional distress; students develop counseling strategies and identify community resources

Year 4: Advanced Pharmacy Practice Experiences (APPEs)
- Integration: Jane's case is revisited during ambulatory care or rheumatology rotations
- Focus: Monitoring disease progression, interdisciplinary collaboration, patient education

Faculty Development and Curriculum Committee Engagement

To implement this educational pathway, faculty can present institutional survey data to the Curriculum Committee to demonstrate educational gaps in SLE preparedness. Proposed changes should align with ACPE standards and emphasize improvements in clinical competence and health equity. Pilot modules may be introduced through electives, co-curricular workshops, or interprofessional education initiatives to build support and assess feasibility.
